# MeInfoText 2.0: gene methylation and cancer relation extraction from biomedical literature

**DOI:** 10.1186/1471-2105-12-471

**Published:** 2011-12-14

**Authors:** Yu-Ching Fang, Po-Ting Lai, Hong-Jie Dai, Wen-Lian Hsu

**Affiliations:** 1 Institute of Molecular and Cellular Biology, National Taiwan University, Taipei, Taiwan; 2 Department of Computer Science, National Chengchi University, Taipei, Taiwan; 3 Institute of Information Science, Academia Sinica, Nankang, Taipei, Taiwan

## Abstract

**Background:**

DNA methylation is regarded as a potential biomarker in the diagnosis and treatment of cancer. The relations between aberrant gene methylation and cancer development have been identified by a number of recent scientific studies. In a previous work, we used co-occurrences to mine those associations and compiled the MeInfoText 1.0 database. To reduce the amount of manual curation and improve the accuracy of relation extraction, we have now developed MeInfoText 2.0, which uses a machine learning-based approach to extract gene methylation-cancer relations.

**Description:**

Two maximum entropy models are trained to predict if aberrant gene methylation is related to any type of cancer mentioned in the literature. After evaluation based on 10-fold cross-validation, the average precision/recall rates of the two models are 94.7/90.1 and 91.8/90% respectively. MeInfoText 2.0 provides the gene methylation profiles of different types of human cancer. The extracted relations with maximum probability, evidence sentences, and specific gene information are also retrievable. The database is available at http://bws.iis.sinica.edu.tw:8081/MeInfoText2/.

**Conclusion:**

The previous version, MeInfoText, was developed by using association rules, whereas MeInfoText 2.0 is based on a new framework that combines machine learning, dictionary lookup and pattern matching for epigenetics information extraction. The results of experiments show that MeInfoText 2.0 outperforms existing tools in many respects. To the best of our knowledge, this is the first study that uses a hybrid approach to extract gene methylation-cancer relations. It is also the first attempt to develop a gene methylation and cancer relation corpus.

## Background

Epigenetics involves the study of mitotically heritable changes in gene expression that are mediated by DNA and histone modifications without altering the DNA sequence [1]. DNA methylation, one of the most critical epigenetic events in mammals, is primarily found on the carbon 5 position of the cytosine ring in the context of CpG dinucleotides [2]. During the methylation reaction, DNA methyltransferases (DNMTs) catalyze the transfer of a methyl group from S-adenosyl-L-methionine (SAM). A number of studies have found that abnormal gene methylation, including hypermethylation and hypomethylation, are associated with the development and progression of cancer; however, the precise mechanisms are still unclear [3,4]. Hence, if the methylation profile is unique to a certain type of cancer, DNA methylation could be an important diagnostic and prognostic biomarker [5].

Information extraction (IE), a field of text mining, selects specific facts about pre-defined types of entities and relationships of interest [6]. As the publication rates in epigenetics have grown exponentially in recent years, several studies have tried to extract information about gene methylation-cancer associations from large collections of textual data. For example, MeInfoText 1.0 uses term co-occurrences in abstracts and sentences together with association rules to identify such relationships [7]. The PubMeth database also uses co-occurrences and the data is reviewed manually to identify cancer-gene methylation associations [8]. The major drawback with co-occurrence methods is that the results may contain a large number of false positive relations due to the lack of syntactic and semantic analysis. Moreover, manual curation may lead to low recall results. As mentioned above, DNA methylation plays an important role in abnormal gene expression and cancer development. However, little research has been done on automatically extraction DNA methylation information with the use of machine learning or other natural language processing techniques [9].

The field of biomedical research is highly versatile [10]. Domain-specific text mining methods must be developed to help researchers and physicians in coping with information overload [11]. In this paper, we present MeInfoText 2.0, which uses a hybrid approach to extract gene methylation-cancer relations. The updated database provides more accurate and large-scale gene methylation profiles as well as the distributions of different types of cancer without the need for a great deal of manual curation. This work would aid epigenetics researchers in making more efficient use of the existing knowledge for practical application.

### Construction and content

**Figure 1:**
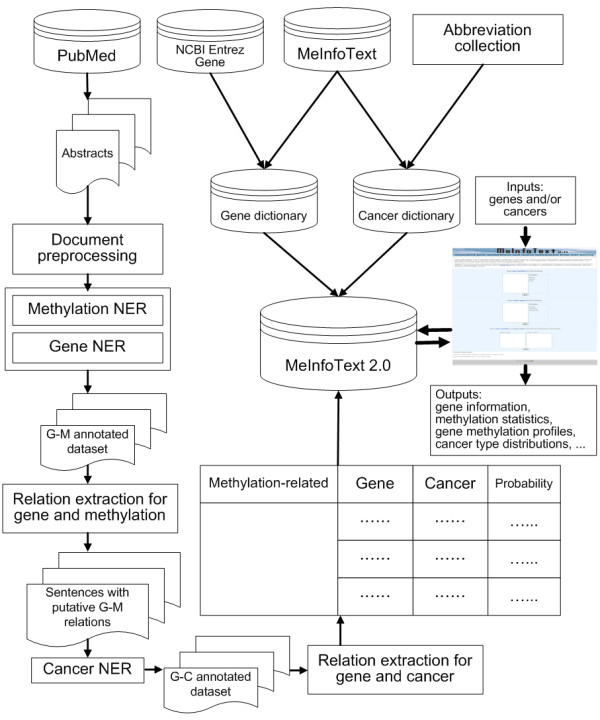
**The system architecture of MeInfoText 2.0**. DNA methylation-related abstracts were collected from PubMed and processed by sentence splitting and expansion. Then, our methylation and gene mention tagger annotated the collected abstracts with methylation terms and gene names, after which the relations between two entities were extracted by two trained maximum entropy (ME) models. The first model determined the gene-methylation (G-M) relation. All positive sentences were annotated with the type of cancer by using a pattern-based cancer type tagger. The second ME model extracted gene-cancer relations. The gene information, methylation statistics and associations ranked in descending order of probability are stored in MeInfoText 2.0. Users can query the database via the web interface using gene names or cancer types, or a combination of both.

MeInfoText 2.0 is a relational database implemented by MySQL 5.0 and PHP programming language. To compile the database, we collected 11,770 abstracts from PubMed by using "human," "methylation" and "cancer" as keywords. From the abstracts, we generated 40,365 sentences containing methylation-related terms. Human gene information, including Entrez Gene ID, official gene symbol, aliases, full name and summary was obtained from NCBI Entrez Gene to construct a gene dictionary. Based on the dictionary, we employed our gene normalization technology [12] to normalize the recognized gene mentions. Figure 1 shows the system architecture of MeInfoText 2.0 and the steps of the information extraction process.

#### Named entity recognition (NER)

Gene symbols/names were identified by NERBio, an ML-based Bio-NER system with an F-score of 85.76% [12-14]. We utilized the pattern "(hyper|hypo)?(-)?(methylat.+)" to identify methylation named entities, where '.' indicates a character and '+' indicates that the character immediately to the left of the symbol may appear more than once.

To develop the cancer named entity recognizer, we combined a cancer dictionary and regular expression patterns. The dictionary containing cancer names was compiled from MeInfoText 1.0 and the abbreviations for different types of cancer were collected from two public websites, http://www.cancerindex.org/medterm/medtm15.htm and http://www.changbioscience.com/abbr/a0a.htm. The patterns used to identify cancer types are as follows:

(1) ABBREVIATION;

(2) (TUMOR SITE) (CANCER-RELATED KEYWORD);

(3) (.+oma|leukemia|leukaemia).

ABBREVIATION includes the acronyms for cancer types such as NPC (nasopharyngeal carcinoma) and CRC (Colorectal Cancer). CANCER-RELATED KEYWORD is a specialized lexicon comprised of the following surface names: cancer, tumor (tumour), neoplasm, carcinogenesis, tumorigenesis and metastasis. With the exception of pattern (1), the matching strategies are case-insensitive

#### Gene methylation-cancer relation extraction

We formulate the task of extracting gene methylation-cancer relations as a binary classification problem. The first model determines if each gene-methylation (G-M) pair in the annotated sentences is positive. Then, the derived positive sentences are input to the second model to identify positive gene-cancer (G-C) pairs.

#### Dataset

Since there are no publicly available annotated corpora for training a gene methylation-cancer association extraction system, we collected epigenetics-related abstracts from MeInfoText 1.0 to compile G-M and G-C corpora. Then we manually annotated each G-M pair and G-C pair in the sentences. If a sentence contained more than one G-M or G-C pair, the sentence was duplicated several times according to the number of possible combinations of the terms in all the pairs so that each sentence only contained one pair. For example, the sentence, **S1**, "**[SOCS1]_gene_**, **[SOCS2] _gene_**, **[RASSF1a] _gene_**, **[CDKN2a]_gene_**, and **[MGMT]_gene _**were **[methylated]_methylation _**in 75, 43, 64, 75, and 64% of **[melanoma]_cancer _**samples, respectively", contains 5 G-M pairs. Therefore, it would be duplicated and rewritten as 5 sentences, each containing exactly one G-M pair for G-M model training. For the G-M corpus, if a gene entity in a sentence is described as methylated, then the relation is regarded as positive; otherwise, it is regarded as negative. In the above sentence, there are five positive instances. For the G-C corpus, if the methylated gene described in a sentence is involved in the development of cancer or the gene's methylation status is detectable in cancers, then the relation is labeled as positive; otherwise, it is labeled as negative. In the above example, five positive G-C pairs were generated. Both corpora contained 1,000 positive and 1,000 negative sentences.

#### Inter-annotator agreement

We randomly selected a subset of 400 sentences from our corpus. Gene, methylation and cancer named entities (NE) and G-M relations and G-C relations were manually annotated by two annotators with biomedical background. The inter-annotator agreement is 95% determined as the intersection of annotated NE and relations divided by the total number of NE and relations.

#### Model generation

**Figure 2:**
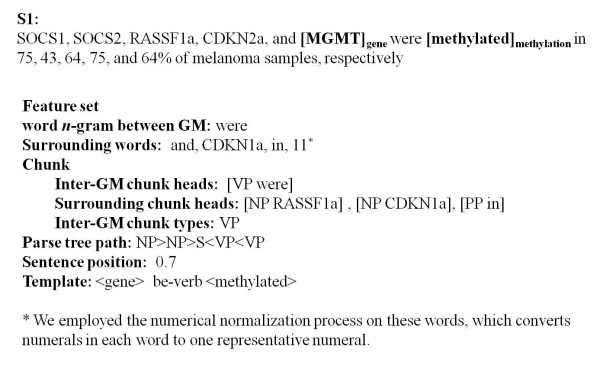
**The employed feature set and extracted feature values for a G-M pair**: word *n*-gram features include all word unigrams and bigrams located between G and M; surrounding word features include the two words before the first named entity and the two words after the second named entity; chunk features include inter-GM chunk heads, surrounding chunk heads and inter-GM chunk types; the parse tree path feature is the syntactic path through the parse tree from the first named entity to the second named entity; and sentence position means the relative position of a sentence in an abstract.

We use the Maximum Entropy (ME) [15] learning model as the ML tool. Figure 2 shows the employed feature set and extracted feature values for the G-M pair, MGMT and methylated, in sentence S1. Our feature set includes the word *n*-grams between entities, surrounding words, chunks, parse tree paths, the relative position of the sentence containing the entities in an abstract, and the template features, which were also used in our previous work for hypertension gene extraction [16]. To generate template features for G-M and G-C, we used Smith and Waterman's local alignment algorithm [17,18] to calculate the similarity of all paired sentences with true associations. The scoring scheme of an award of +1 for match and a penalty of -1 for both mismatch and gap was adopted. Then, we manually reviewed the paired sentences with the top 100 scores to create templates, such as " < gene > promoter < methylation > " and " < gene > is frequently methylated in < cancer > ". We used Zhang's MaxEnt toolkit [19] to train two ME models, denoted as GM and GC, for G-M and G-C relation extraction respectively.

### Utility

#### Query method

**Figure 3:**
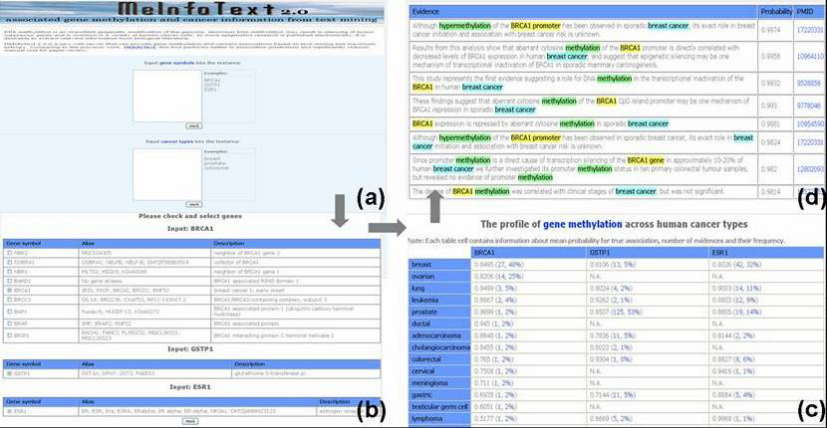
**The web interface of MeInfoText 2.0**. Figure 3 (a) the search interface with three search options: (1) by inputting gene names, (2) by selecting cancer types, and (3) by combining specific genes and types of cancer. After checking the query terms, the system returns the gene methylation profile across human cancer types and highlights the evidence sentences, as shown in Figures 3 (c) and 3(d).

Figure 3 shows the MeInfoText 2.0 web interface, which can be accessed by inputting a gene name or type of cancer, or a combination of the two. Searching by genes yields general gene information, cross-references, gene methylation statistics and cancers related to the gene methylation ranked by the following equation:

(1) $$\text{Ranking}\;\text{score}=\overset{n}{\underset{i=1}{\sum }}P{\left(\text{Gene}\;\text{X}\;\text{Methylation},\;\text{Cancer}\;\text{Y}\right)}_{i\text{th}\;\text{Sentence}}$$

For each G-C pair, there may be more than one evidence sentence with the relation probability calculated by the GC maximum entropy model. The sum of total probabilities for each gene and cancer relatedness is used to represent the ranking score of the G-C pair. Querying by cancer type returns genes methylated in the cancers ranked in the same way. Users can also find genes methylated in pre-specified cancer types by searching genes and cancers together. The relations extracted from the current literature are also available. In this study, the profiles of gene methylation across human tumor types provide the frequency patterns of gene methylation based on the number of evidence sentences and the maximum probability, as shown in Figure 3 (c).

For example, to access information about the BRCA1 (breast cancer 1, early onset), GSTP1 (glutathione S-transferase pi) and ESR1 (estrogen receptor 1) gene methylation profile across various types of cancer, a user could input BRCA1, GSTP1, ESR1 as search terms separated by line breaks. Gene orthographic variants, such as BRCA1 and BRCA-1, are generated based on a few simple rules [20]. Next, the system checks if the searched genes are available in the database. After selecting gene symbols, the returned web page displays a table containing information about gene-cancer pairs, the average maximum probability, the number of evidence sentences and their frequency. The average maximum probability is the sum of the ranking scores divided by the number of sentences. The cancer set shown in the left-hand column lists the cancers associated with any one of the three genes. The user can then examine the extracted sentences ranked by their maximum probability scores. Keywords are highlighted and links to PubMed are also provided. In addition to the gene methylation profile, if only a single gene is queried, the gene summary, cross-references to other public databases, and statistics about hypermethylation and hypomethylation are shown.

Users can select one type of cancer, such as breast cancer, (or several types) to find a set of genes undergoing abnormal methylation related to the cancer(s) of interest. It is also possible to input multiple official gene symbols separated by line breaks and select multiple cancer types to retrieve the profiles of gene methylation across human cancer types. For instance, let us consider the following set of genes discussed by Esteller et al. [21]: CDKN2A, CDKN2B, MGMT (O6-methylguanine-DNA methyltransferase), MLH1, BRCA1, GSTP1, DAPK1, CDH1 (cadherin 1), TIMP3 (TIMP metallopeptidase inhibitor 3), TP73, and APC (adenomatous polyposis coli). If the genes are input to the system and different types of cancer, such as colorectal, lung, breast, brain, gastric, liver, esophageal, bladder, blood, kidney, ovarian, head and neck, pancreatic, endometrial and lymphatic cancer, are selected, the methylation profile of each gene related to the different cancers will be shown. The profile may also reflect the specific involvement of the gene in the selected type of cancer or groups of cancers.

## Discussion

The pages retrieved by MeInfoText 2.0 for the above gene methylation profile are similar to the results reported by Esteller et al. [21]. For example, CDKN2A is hypermethylated across colorectal, lung and breast cancer with an average maximum probability of 0.8 and a total of 306 evidence sentences. Meanwhile, hypermethylation of BRCA1 is found primarily in breast and ovarian cancer with an average maximum probability of 0.84 and approximately 41 evidence sentences.

In the next section, we consider the NER performance as well as the G-M and G-C relation extraction performance. The predictive performance measures for the trained models are defined as follows: $\text{precision}=\frac{TP}{TP+FP}$ and $\text{recall}=\frac{TP}{TP+FN}$, where *TP*, *FP *and *FN *denote the number of true positives, false positives and false negatives respectively.

### NER and relation extraction performance

The data used to evaluate the cancer named entity recognizer was downloaded from http://biotext.berkeley.edu/data/dis_treat_data/sentences_with_roles_and_relations[22]. The disease named entities tagged <DIS></DIS> or <DISONLY></DISONLY> and the cancer names, except general terms like tumor, were used for evaluation. If an exact matching strategy is employed, the precision and recall rates for cancer name recognition are 85.2% and 79.5% respectively; while under the approximate matching strategy [23], the rates are 99.1% and 81.8% respectively.

To evaluate G-M and G-C relation extraction, we applied 10-fold cross validation on the G-M and G-C corpora. We randomly selected 900 sentences from the corpora to train the GM and GC models and used the remainder for testing. The average precision/recall rates were 94.7 ± 2.1/90.1 ± 2.8 and 91.8 ± 3.2/90.0 ± 1.6% for the GM and GC models respectively. Both models were trained with all the features shown in Figure 2. There was no obvious performance improvement (< 0.01) when the template features were used.

### System evaluation and utility

We conducted three experiments to evaluate the proposed relation extraction scheme. The first experiment was designed to determine if MeInfoText 2.0 outperformed version 1.0 in terms of extracting gene methylation-cancer relations. We retrieved 403 gene methylation and cancer associations from MeInfoText 1.0 based on the following 20 genes: PYCARD, CDH13, COX2, DAPK1, ESR1, GATA4, SYK, MLH1, TP73, PRDM2, PGR, SFRP1, SOCS1, SOCS3, STK11, TMEFF2, THBS1, RASSF5, PRKCDBP and RARB. The above genes are known to be methylation-related and were used in our previous study. Table 1 shows that our ME models significantly improve the precision of relation extraction at the expense of a small reduction in recall. In the table, Co-occurrences in abstracts and Co-occurrences in sentences refer to the association mining methods used in MeInfoText 1.0.

**Table 1:** Gene methylation and cancer relation extraction precision/recall rates

	Precision (%)	Recall (%)
Co-occurrences in abstracts	62.8	98.8

Co-occurrences in sentences	84.6	100

GM+GC	95.4	90.9

GM	92.4	94.8

The second experiment compared our system's performance with that of PubMeth on an evaluation dataset used by PubMeth in a review article on DNA methylation in breast cancer. The article lists 39 genes known to be hypermethylated in breast cancer. The MeInfoText 2.0 database contains 38 of those genes, and 23 are described in breast cancer. In addition, querying MeInfoText 2.0 for breast cancer information returns more than 150 genes, 87 of which have at least two references and 63 have one reference with a probability greater than 0.8. The review also lists 8 genes reported to be hypomethylated in breast cancer; 7 of them are available in our database and 4 are described in breast cancer. However, PubMeth can not provide hypomethylation information. Table 2 compares the number of genes available, the genes methylated in breast cancer, and the genes searched for breast cancer in the two databases.

**Table 2:** Comparison of the information available in MeInfoText 2.0 and PubMeth

	MeInfoText 2.0	PubMeth
# hypermethylated genes available	38 (97%)	27 (69%)

# hypermethylated genes described in breast cancer	23 (59%)	20 (51.3%)

# hypomethylated genes available	7 (87.5%)	0

# hypomethylated genes described in breast cancer	4 (50%)	0

# genes searched for association with breast cancer	> 150	94

We also compared the search results in terms of the number references and evidence sentences. Searching BRCA1 in PubMeth returned 7 related types of cancer, namely, breast, ovarian, lung, gastric, cervical, pancreatic, and brain cancer. The number of references/evidence sentences for the 7 types of cancer in PubMeth and MeInfoText 2.0 were, respectively, 12/7 vs. 14/27, 19/12 vs. 6/14, 1/0 vs. 2/3, 1/1 vs. 1/1, 1/1 vs. 1/1, 2/1 vs. 0/0, and 1/1 vs. 1/1. Furthermore, we performed a cancer-centric search to find the top 10 breast cancer and gene pairs listed on the PubMeth results page. In Table 3, we compare the association evidence sentences extracted by PubMeth and MeInfoText 2.0. The results demonstrate that MeInfoText 2.0 provides more accurate association information, as well as more references and evidence sentences because of automatic text mining. In addition, the low average reference intersection between PubMeth and MeInfoText 2.0 suggests that the two systems may be complementary.

**Table 3:** Comparison of the cancer-gene methylation association evidence sentences extracted by PubMeth and MeInfoText 2.0

	PubMeth # references	PubMeth # evidence sentences	MeInfoText2.0 # references	MeInfoText2.0 # evidence sentences	Reference intersection (%)
(breast, BRCA1)	11	7	14	27	2 (18.2%)
(breast, RASSF1)	10	9	20	27	6 (60%)
(breast, CDKN2A)	10	7	25	45	3 (30%)
(breast, APC)	11	10	10	17	3 (27.3%)
(breast, CDH1)	10	8	12	14	2 (20%)
(breast, GSTP1)	4	3	7	12	0 (0%)
(breast, ESR2)	5	4	3	7	1 (20%)
(breast, CCND2)	7	5	8	11	3 (42.9%)
(breast, ESR1)	5	5	15	22	1 (20%)
(breast, SFRP1)	2	5	6	11	2 (100%)

The third experiment was designed to further evaluate the performance of MeInfoText 2.0 using data published by Kim et al, 2010 [24], which reports 58 genes methylated in colorectal cancer (CRC). All 58 genes are available in our database, and 72.4% are reported to be associated with gene methylation and CRC. The average maximum probability is 0.87 and there are 10 evidence sentences. The second and third experiments indicate that MeInfoText 2.0 performs well in epigenetics studies that focus on different types of cancer.

Veeck and Esteller [4] identified hypermethylation as an important mechanism in miRNA silencing; and they listed 3 miRNAs silenced by, or involved in, epigenetic mechanisms. Of the 3 genes, it has been reported that miR-9-1 with unknown target genes is hypermethylated in human breast cancer [25], an association also found by MeInfoText 2.0. Information about tumorgenesis and miRNA gene methylation, such as miR-34a [26] and miR-181c [27], is also available in the database. Moreover, the extracted information shows that aberrant expression of DNMT1 (DNA methyltransferase 1) breast cancer is associated with the loss of DNA methylation [28]. To determine if DNMT1 or other DNA methyltransferases may be the potential target genes of miR-9-1, we used microRNA.org [29] for prediction. Our database does not contain any miR-9-1 information, but it can retrieve the prediction results for miR-9. Although DNMT1 is not predicted as the target gene of miR-9, microRNA.org shows that MeCP2 (methyl CpG binding protein 2) may contain two hsa-miR-9 target sites. A previous study [30] posited that the silence of the ER promoter in the breast cancer cell line is associated with DNA hypermethylation, histone modification and the recruitment of MeCP2, DNMT1 and other proteins. The hypothesis suggests that miR-9-1 hypermethylation abnormally increases the expression of MeCP2, which in turn represses the transcription of methylated DNA via the recruitment of a histone deacetylase activity associated with DNMT1 [31]. Further investigation is needed to elucidate the relationships between miR-9-1, MeCP2 and DNMT1 in breast carcinogenesis.

## Conclusion

MeInfoText 2.0 provides more accurate information about gene methylation-cancer associations discussed in a large number of studies. To the best of our knowledge, this is the first study that uses machine learning, a domain dictionary and pattern matching to extract genetic-epigenetic relations. Such relations are important for determining if unique profiles exist for specific types of cancer, and assessing how to improve cancer detection and treatment by using DNA methylation biomarkers. The study is also the first attempt to create a gene methylation-cancer corpus.

## Availability and requirements

Project name: MeInfoText 2.0

Project home page: http://bws.iis.sinica.edu.tw:8081/MeInfoText2/

Operating system(s): platform independent

License: the database website is freely accessible

## Authors' contributions

YCF compiled the database, developed the web server, designed and conducted all the experiments, annotated the corpora, and drafted the manuscript. PTL helped construct the maximum entropy models for relation extraction. HJD and WLH provided constructive opinions and refined the manuscript. All authors read and approved the final manuscript.
